# A taxonomic study on Epedanidae from Thailand including functional aspects of male genital morphology (Opiliones, Laniatores)

**DOI:** 10.3897/zookeys.915.47626

**Published:** 2020-02-24

**Authors:** Chao Zhang, Jochen Martens

**Affiliations:** 1 The Key Laboratory of Invertebrate Systematics and Application, College of Life Sciences, Hebei University, Baoding, Hebei 071002, China Hebei University Baoding China; 2 Institut für Organismische und Molekulare Evolutionsbiologie, iomE, D-55099 Mainz, Germany Institut für Organismische und Molekulare Evolutionsbiologie Mainz Germany; 3 Senckenberg Research Institute, Arachnology, D-60325 Frankfurt am Main, Germany Senckenberg Research Institute Frankfurt am Main Germany

**Keywords:** Arachnida, harvestmen, male genitalia, taxonomy

## Abstract

The South-East Asian opilionid family Epedanidae Sørensen, 1886 has one of its strongholds in Thailand from where a multitude of genera and species have been described but the epedanid fauna of the country is still poorly known. This paper records four species from this country, three of which are new: *Euepedanus dashdamirovi***sp. nov.** (male and female), *Plistobunus jaegeri***sp. nov.** (male and female), and *Toccolus kuryi***sp. nov.** (male and female). *Toccolus globitarsis* Suzuki, 1969 was previously known only from the type locality in Thailand and is redescribed here. Functional aspects of epedanid penial morphology are highlighted.

## Introduction

The opilionid family Epedanidae Sørensen, 1886 is widespread in the Indo-Malay Realm where it contains many genera, but the local species are often narrowly distributed and still poorly known. Thailand is one of the strongholds of the family. Many epedanid species display a bizarre peripheral morphology with heavily armed chelicerae, pedipalps, and a surprisingly long pointed spine or apophysis on top of the ocularium. In many cases, genera within the Epedanidae are not well characterized and probably do not represent monophyletic entities. This paper aims at increasing knowledge of the Thai fauna in re-defining three known genera and one known species, and in proposing three new species.

The two Epedanidae species first described from Thailand are *Euepedanus orientalis* (Hirst, 1912) and *Caletorellus siamensis* (Hirst, 1912). Originally, these were placed in genus *Epedanus* Thorell, 1876. Subsequently, *E. orientalis* was transferred to *Mimepedanus* Roewer, 1923 by Roewer (1923) and this genus was synonymized with *Euepedanus* Roewer, 1915 by Suzuki (1969b). The second species, *E. siamensis* was transferred to *Euepedanus* by Roewer (1923), and later to *Caletorellus* Roewer, 1938 by Roewer (1938) and is the type species of the latter genus.

Roewer (1938) systematically reorganized what is today known as Epedanidae (for him they were all included in Phalangodidae Simon, 1879) and he erected three subfamilies in it: Acrobuninae Roewer, 1912, Epedaninae Roewer, 1912 (incorrectly stated, author of subfamily is Sørensen 1886), and Sarasinicinae Roewer, 1912. Suzuki subsequently published three new Thai epedanid genera, and ten new species (Suzuki 1969a, 1969b, 1981, 1985; Suzuki and Stone 1986). All these descriptions are very accurately carried out including meticulous drawings of relevant characters of male genital traits.

The Naturmuseum Senckenberg, Frankfurt am Main, Germany houses most of Roewer's Epedanidae types which were described by him over several decades (Roewer 1912, 1915, 1927,1938), and this institution is continuously enlarging its collections. In this paper, we study a small epedanid collection from Thailand, which was gathered in 2010 by Selvin Dashdamirov. In addition, we pay attention not only to epedanid male genital morphology for species recognition but in addition try to disentangle functional morphology of the movable parts of the copulatory organs.

## Materials and methods

Taxonomic methods follow the outline proposed by Acosta et al. (2007). The specimens were preserved in 70% ethanol, and examined and drawn under a Leica M205A stereomicroscope equipped with a drawing tube. Further details were studied using a compound microscope (Nikon YS100). Photographs were taken using a Leica M205A stereomicroscope equipped with a DFC450 CCD. The male genitalia were placed first in hot lactic acid, later transferred to distilled water to expand the movable parts of glans penis for observation (Schwendinger and Martens 2002). The terminology of genital structures follows Martens (1986) and Macías-Ordóñez et al. (2010), the macrosetae terminology follows Kury and Villarreal (2015). The type specimens are deposited in the Senckenberg Arachnology collections (SMF).

All measurements are given in mm. The following abbreviations are used in the text:

**CI** capsula interna;

**CE** capsula externa;

**Pb** pars basalis;

**Pd** pars distalis;

**S** stylus;

**VP** ventral plate.

## Taxonomy

### Euepedanus

Taxon classificationAnimaliaOpilionesEpedanidae

Roewer, 1915

88CD4B9E-2C14-5311-B0B6-64346017257E

Euepedanus Roewer 1915: 62; 1923: 206 (in part); 1938: 114. Suzuki 1969b: 83; 1985: 81. Zhu and Lian 2006: 64.
Mimepedanus Roewer 1923: 208; 1938: 105. Synonymized by Suzuki 1969b: 83.

#### Type species.

*Euepedanus trispinosus* Roewer, 1915, subsequent designation by Suzuki (1969b: 83).

#### Diagnosis.

Medium-sized epedanines (3.00–5.00 mm) with a long median spine on the ocularium. Area II with a pair of long spines. The basichelicerite of chelicerae with numerous tubercles. Pedipalpal femur ventrally with 5–7 seta-tipped tubercles, and distally with two tubercles on medial side. Pedipalpal patella with at least three seta-tipped tubercles. Distitarsus of leg I with two segments (cf. Zhu and Lian 2006: 64).

#### Included species

(nine species). *Euepedanus trispinosus* Roewer, 1915, *Euepedanus orientalis* (Hirst, 1912), *Euepedanus chaiensis* Suzuki, 1969b, *Euepedanus dividuus* Suzuki, 1969b, *Euepedanus similis* Suzuki, 1985, *Euepedanus pentaspinulatus* Suzuki, 1985, *Euepedanus spinosus* Suzuki, 1985, *Euepedanus flavimaculatus* Zhu and Lian, 2006, and *Euepedanus dashdamirovi* sp. nov.

#### Distribution.

Thailand, China, Malaysia, Singapore.

### Key to the currently known species of *Euepedanus*

**Table:** 

1	Anterior margin of prosoma unarmed	**2**
–	Anterior margin of prosoma armed with tubercles	**4**
2	Basichelicerite of chelicerae laterally and medially with prominent spines	***E. trispinosus* (Singapore and Malaysia)**
–	Basichelicerite of chelicerae laterally and medially without prominent spines, with small tubercles or teeth	**3**
3	Pedipalpal femur ventrally with five seta-tipped tubercles	***E. similis* (Thailand)**
–	Pedipalpal femur ventrally with seven seta-tipped tubercles	***E. flavimaculatus* (China)**
4	Femora of all legs with ventral row of seta-tipped granules	***E. spinosus* (Thailand)**
–	Femora of all legs without a ventral row of seta-tipped granules	**5**
5	Scutal area I divided into three parts	***E. dividuus* (Thailand)**
–	Scutal area I entire	.**6**
6	Posterolateral angle of scutum with one large tubercle	***E. orientalis* (Thailand)**
–	Posterolateral angle of scutum without one large tubercle	**7**
7	Conspicuous spines dorsally on the basichelicerite of chelicerae dispersed evenly	***E. dashdamirovi* sp. nov. (Thailand)**
–	Conspicuous spines dorsally on the basichelicerite of chelicerae dispersed unevenly, gathered near the base	**8**
8	Median tubercles of free tergites I and II enlarged, cheliceral hand with a ventral seta-tipped tubercle	***E. pentaspinulatus* (Thailand)**
–	Median tubercles of free tergites I and II small, cheliceral hand without a ventral seta-tipped tubercle	***E. chaiensis* (Thailand)**

### Euepedanus dashdamirovi sp. nov.

Taxon classificationAnimaliaOpilionesEpedanidae

D0255543-8960-5464-A271-966AE7217187

http://zoobank.org/8A17DBE6-BBA9-474B-B7CB-03867E526C5B

Figures 1–8 , 9–17 , 18–25 , 26–31

#### Type material.

***Holotype*** male (SMF-CJM7059-01): THAILAND: Sa Kaeo (Kaew) Province: Ta Phraya District, Ta Phraya NP (No.11), 14°8.37'N 102°40.19'E, alt. 164 m, 27 October 2010, S. Dashdamirov leg. ***Paratypes.*** one female (SMF-CJM7059-02), same collecting data as holotype; one female (SMF-CJM7051-01) and one juvenile (SMF-CJM7051-02), THAILAND: Sa Kaeo (Kaew) Province: Maeng, Srakaeo District, Wat Tham Khao Maka (No.8), 13°47.23'N 101°56.87'E, alt. 46m, 26 October 2010, S. Dashdamirov leg.

#### Diagnosis.

Penis with a stereoscopic ventral plate (e.g., Fig. 132) and the stylus constricted apically. Anterior margin of prosoma armed with tubercles. The basichelicerite of chelicerae dorsally with five tubercles (Figs 2–4). Pedipalpal femur ventrally with six seta-tipped tubercles, and distally with two tubercles on medial side. Pedipalpal patella with three seta-tipped tubercles.

#### Etymology.

Named in honor of the collector of the type material and our good friend, Dr. Selvin Dashdamirov.

#### Description.

**Figures 1–8.**
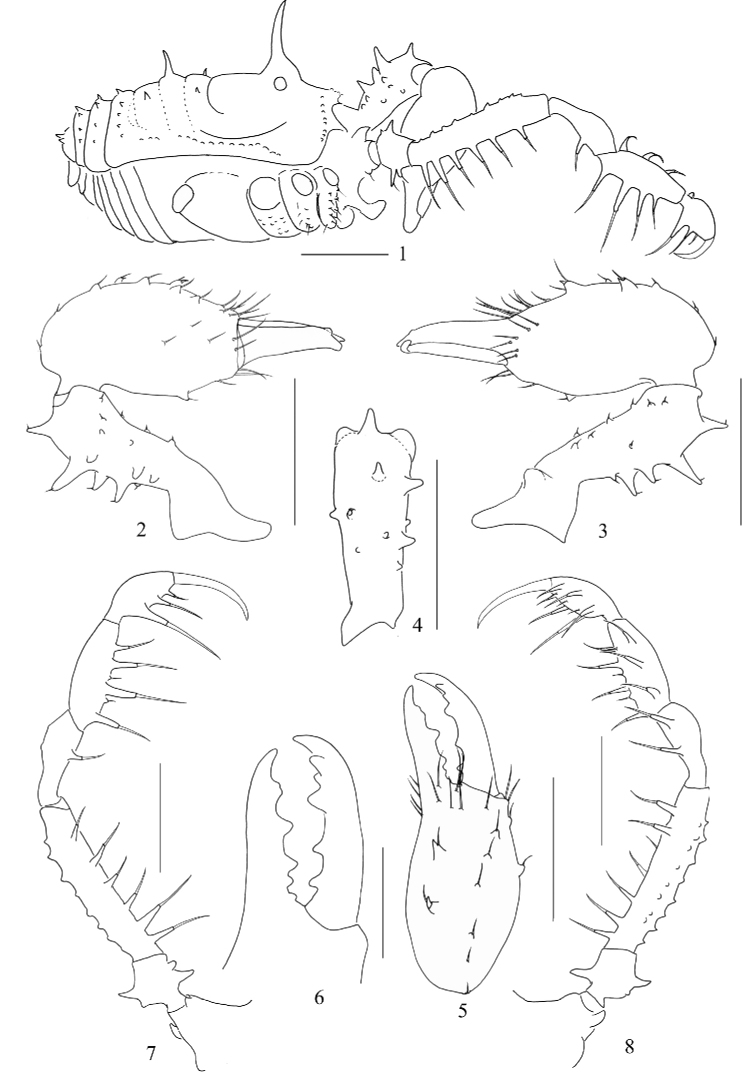
*Euepedanus dashdamirovi* sp. nov., male holotype **1** body, lateral view **2** right chelicera, lateral view **3** same, medial view **4** right basichelicerite, dorsal view **5** right cheliceral hand, frontal view **6** Right cheliceral fingers, frontal view **7** right pedipalp, lateral view **8** same, medial view. Scale bars: 1 mm (**1–5, 7–8**), 0.5 mm (**6**).

**Figures 9–17.**
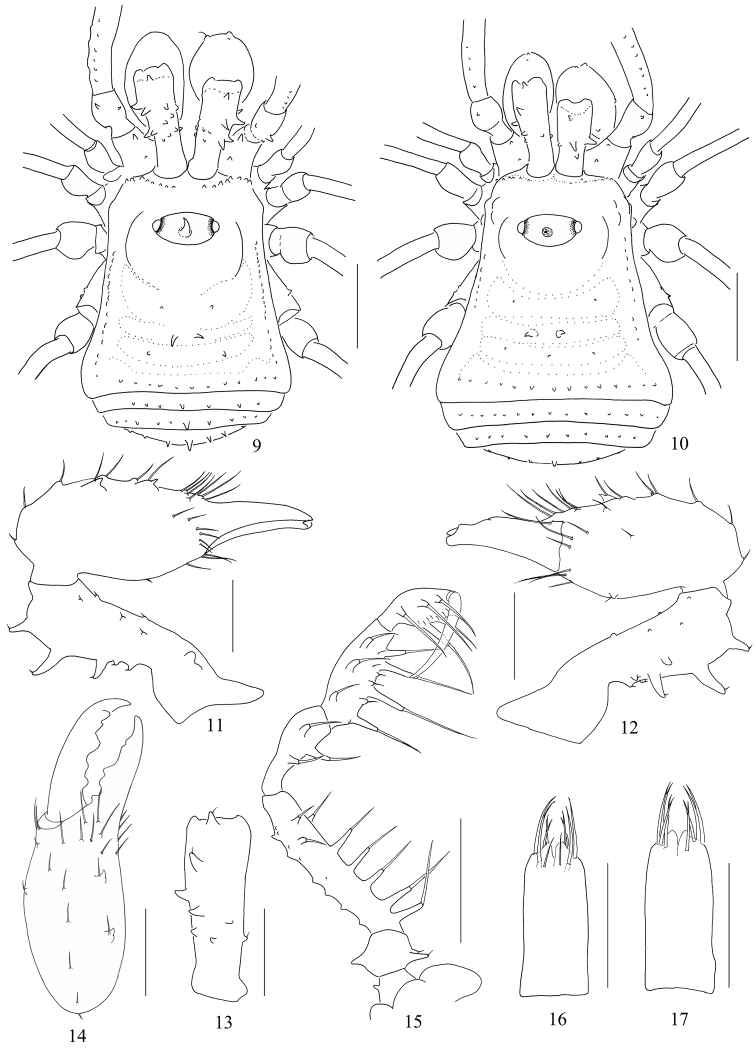
*Euepedanus dashdamirovi* sp. nov., male (Fig. 9, holotype), female (Figs 10–17, paratype) **9** male body, dorsal view **10** female body, dorsal view **11** right chelicera, medial view **12** same, lateral view **13** left basichelicerite, dorsal view **14** left cheliceral hand, frontal view **15** left pedipalp, medial view **16** ovipositor, ventral view **17** same, dorsal view. Scale bars: 1 mm (**9, 10, 15**), 0.5 mm (**11–14, 16, 17**).

Male (holotype) habitus as in Figs 1, 9, 26, 27. Coloration (Figs 26, 27): entire body dorsally rusty yellow with black patches; median area of prosoma with black reticulations before and behind the ocularium; both lateral ridges of scuta with brown stripes; opisthosomal area I bordered posteriorly with brown and area II dark brown with paler interspaces; areae III and IV and free tergites each with a transverse dark band; venter concolorous with the dorsum; chelicerae and pedipalps reticulated; all leg segments other than trochanters and tarsi with black reticulations.

***Dorsum*** (Figs 9, 26). Dorsal scutum more or less trapezoid in form, with sides slightly curved, proximal end of abdomen slightly enlarged and rounded. Anterior margin of prosoma with eight small tubercles at the lateral portion. Ocularium long oval, removed from anterior border of scutum by 0.50 mm, armed with an elongate, lightly curved spine. Posterior margin of prosoma bowed. Opisthosomal area I longer than other areae. Areae I and III each with a median pair of short spines. Area II with two median long spines. Area IV and all free tergites with a transverse row of seta-tipped tubercles, central ones larger than others, like slender spines. Lateral margins of the scutum with a longitudinal row of seta-tipped tubercles.

***Venter*** (Fig. 28). Coxae with tubercular surface, coxa I with enlarged seta-tipped tubercles. Coxae II, III, and IV with only minute similar tubercles. Genital operculum with a few seta-tipped granules. Free sternites with a row of minute tubercles, each with setae on top. Spiracles clearly visible.

***Chelicerae*** (Figs 2–6). Basichelicerite elongate, dorsally with a slight bulla, and armed with four long spines and one seta-tipped tubercle dorsally; two similar spines and one seta-tipped tubercle medially on the ectal side; one seta-tipped tubercle on the lateral side; ten seta-tipped tubercles scattered over the ventral side; medial side with a basal protuberance. Cheliceral hand widened, pro-dorsally with some seta-tipped tubercles; two seta-tipped tubercles on the ventral side. Fingers relatively strong, inner edges toothed (Fig. 6): moveable finger and fixed finger each with five stump teeth.

***Pedipalps*** (Figs 7, 8). Coxa dorsally with two seta-tipped tubercles; ventrally with one distal seta-tipped tubercle. Trochanter ventrally and dorsally each with one enlarged and one small seta-tipped tubercles. Femur ventrally with a row of six seta-tipped tubercles, of which the two longest at the base; dorsally with two longitudinal rows of conical tubercles; distally on medial side with two seta-tipped tubercles. Patella ventro-mesally with two setiferous tubercles, and ventro-ectally with one setiferous tubercles. Tibia ventro-mesally with three setiferous tubercles, and ventro-ectally with four setiferous tubercles. Tarsus with three setiferous tubercles on each side of ventral side. Tarsal claw curved, longer than tarsus.

***Legs.*** All segments smooth, only with scarce short setae. Femur IV slightly curved. Distitarsus I with two, distitarsus II with three tarsomeres. Distitarsi III and IV without scopula. Tarsal claws smooth. Tarsal formula (I–IV): 9(2)/24(3)/7/8.

**Figures 18–25.**
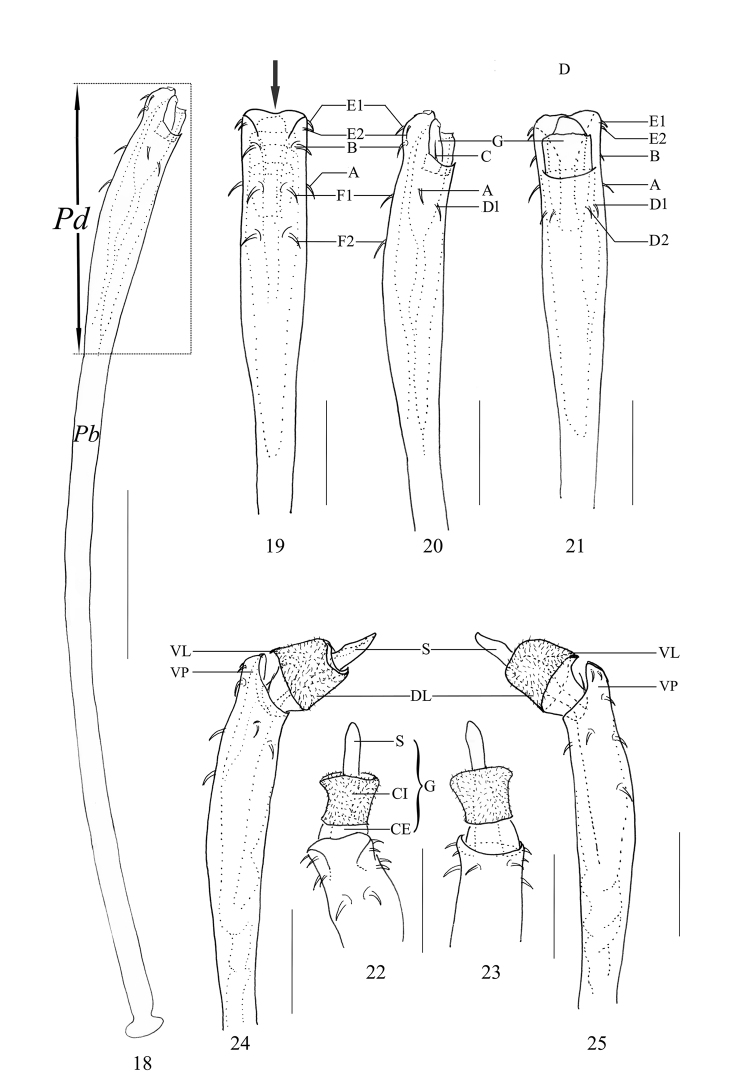
*Euepedanus dashdamirovi* sp. nov., genitalia of male holotype **18** penis, lateral view **19** pars distalis of penis, ventral view (arrow indicates indented distal end) **20** same, lateral view **21** same, dorsal view **22** pars distalis of penis (everted), ventral view **23** same, dorsal view **24–25** same, lateral view. Abbreviations: **CE** capsula externa **CI** capsula interna **DL** dorsal lobe **G** glans **Pb** pars basalis **Pd** pars distalis **S** stylus **VP** ventral plate **VL** ventral lobe. Scale bars: 0.5 mm (**18**), 0.25 mm (**19–25**).

***Penis*** (Figs 18–25). Shaft slender, elongate. The distal fourth of the shaft broadened and nearly parallel-sided till apical portion (pars distalis). Ventral plate nearly square (ventral view, Fig. 19), and slightly indented on the distal margin (Fig. 19). Glans partially sunken into dorsal depressed portion of pars distalis and not extending the distal margin of the ventral plate. Capsula externa and capsula interna cylindrical, and the inner side of capsula interna with dense cover of fur-like microtrichia. The ventral lobe of capsula interna conspicuous, somewhat triangular; the dorsal lobe inconspicuous. Stylus with irregular shape, constricted apically. Spination symmetrical. One pair of setae A, B, and C. Two pairs of setae D, E, and F (Figs 19–21).

***Female*** (Figs 10–17, 29–31). In general appearance similar to the male, abdomen more rounded posteriorly (Figs 10, 29). Basichelicerite dorsally with three and ectally with one long spines, other seta-tipped tubercles on chelicerae less stout than those of the male (Figs 11–13). Cheliceral hand prodorsally with two enlarged seta-tipped tubercles in the middle of inner portion (Fig. 11); one seta-tipped tubercle on the ventral side (Fig. 12). Pedipalp similar to that of male (Fig. 15). Tarsal formula (I–IV): 8(2)/19(3)/7/8.

***Ovipositor*** (Figs 16, 17). Elongate. Ventral side with four and dorsal side with six setae of different lengths.

#### Measurements.

Male holotype (female paratype): body 2.94 (2.36) long, 2.13 (2.48) wide at widest portion, scutum 2.36 (2.05) long. Ocularium 0.40 (0.30) long, 0.79 (0.69) wide. Proximal chelicerae 1.24 (1.08) long, 0.43 (0.38) wide; second 2.10 (1.72) long, 0.72 (0.59) wide; distal 0.91 (0.80) long, 0.21 (0.20) wide. Pedipalp claw 0.84 (0.80) long. Penis 1.67 long. Measurements of pedipalp and legs as in Tables 1 and 2.

**Table 1.** *Euepedanus dashdamirovi* sp. nov. Measurements of the pedipalp and legs of the male holotype, as length/width.

	**Trochanter**	**Femur**	**Patella**	**Tibia**	**Metatarsus**	**Tarsus**	**Total**
Pedipalp	0.59/0.39	1.65/0.31	0.85/0.30	0.92/0.35		0.57/0.33	4.58
Leg I	0.30/0.27	2.10/0.19	0.51/0.26	1.49/0.14	2.60/0.05	1.30/0.04	8.30
Leg II	0.32/0.33	3.54/0.18	0.64/0.27	3.02/0.15	4.24/0.05	3.22/0.04	14.98
Leg III	0.52/0.44	2.65/0.26	0.65/0.35	1.59/0.23	3.30/0.13	1.30/0.07	10.01
Leg IV	0.45/0.40	3.88/0.28	0.72/0.38	2.15/0.23	4.74/0.09	1.45/0.07	13.39

**Table 2.** *Euepedanus dashdamirovi* sp. nov. Measurements of the pedipalp and legs of the female paratype, as length/width.

	**Trochanter**	**Femur**	**Patella**	**Tibia**	**Metatarsus**	**Tarsus**	**Total**
Pedipalp	0.46/0.32	1.32/0.30	0.72/0.29	0.76/0.34		0.45/0.31	3.71
Leg I	0.33/0.26	1.83/0.17	0.52/0.23	1.26/0.16	2.31/0.03	0.84/0.03	7.09
Leg II	0.34/0.29	2.77/0.16	0.68/0.25	2.82/0.15	3.37/0.08	2.44/0.06	12.42
Leg III	0.34/0.41	2.33/0.26	0.62/0.38	1.49/0.23	2.87/0.06	1.02/0.06	8.67
Leg IV	0.37/0.37	3.42/0.24	0.69/0.33	1.97/0.21	4.28/0.11	1.43/0.05	12.16

#### Habitat.

The specimens were collected by hand under the decaying bark of various tree species.

#### Distribution.

Known only from the type locality.

**Figures 26–31.**
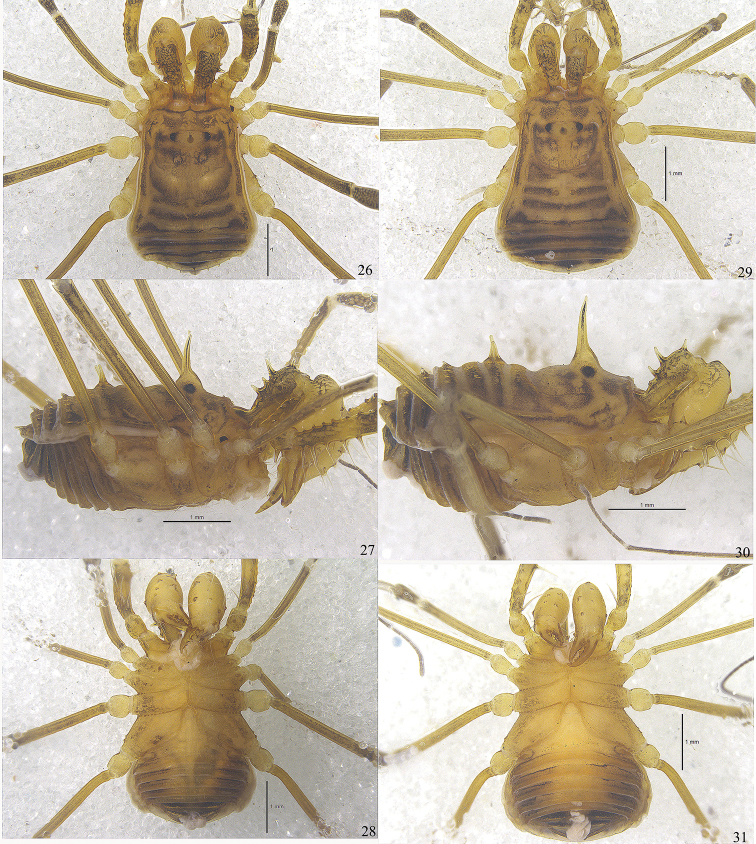
Photographs of male (Figs 26–28, holotype) and female (Figs 29–31, paratype) of *Euepedanus dashdamirovi* sp. nov. **26, 29** body and parts of appendages, dorsal view **27, 30** body and parts of appendages, lateral view **28, 31** body and parts of appendages, ventral view. Scale bars: 1 mm.

### Plistobunus

Taxon classificationAnimaliaOpilionesEpedanidae

Pocock, 1903

9000C3A3-306A-5DB2-B9BF-6DBB8F758C25

Plistobunus Pocock 1903: 447; Roewer 1912: 232; 1923: 207; 1938: 124; Lian et al. 2011: 41.

#### Type species.

*Plistobunus rapax* Pocock, 1903, by original designation.

#### Extended diagnosis.

Medium-sized epedanines (2.40–3.57 mm) with a long median spine on the ocularium. Prosoma with a row of 4–6 setiferous tubercles on each side of the frontal margin. Areae II and IV with or without long spines. The basichelicerite of chelicerae elongate and armed with numerous tubercles, dorsally with three in males and with two in female (Lian et al. 2011: 42, figs 2, 3; 44: 9, 10; 46: 18, 19. Figs 33–35, 47–49). Pedipalpal femur of male ventrally with 9–13 seta-tipped tubercles, and distally with one or two tubercles on medial side. Pedipalpal patella with at least three seta-tipped tubercles. Distitarsus of leg I with two segments. The distal part of pars basalis of penis widened and with a stereoscopic ventral plate. Stylus columnar. Capsula interna ventrally and capsula externa dorsally surround the stylus.

#### Included species.

*Plistobunus rapax* Pocock, 1903, *Plistobunus columnarius*Lian et al., 2011, and *Plistobunus jaegeri* sp. nov.

#### Distribution.

China (Hong Kong and Hainan Island), Thailand (Ubon Ratchathani).

#### Sexual dimorphism.

The species of this genus possess conspicuous sexual dimorphism in the armature of the appendages. The tubercles or spines in male on the basichelicerite of chelicerae, and those of the femur of pedipalps and legs are much more robust or conspicuous than those of in female; the shape of body is very similar in both sexes.

The numbers of seta-tipped tubercles distally on medial side of pedipalpal femur gives an impression of the differences between the species. Male specimens of *P. rapax* and *P. columnarius* are described with two (Lian et al. 2011: 42, fig. 6; 44, fig. 14) and those of *P. jaegeri* sp. nov. with only one such tubercle (Fig. 38). Females of *P. columnarius* (Lian et al. 2011: 46, fig. 22) and *P. jaegeri* sp. nov. (Fig. 52) possess no tubercles of this size or position (female of *P. rapax* unknown).

### Key to the currently known species of *Plistobunus*

**Table:** 

1	Distributed in Thailand, the scutal area without long spines in male	***P. jaegeri* sp. nov.**
–	Distributed in China, scutal area II with a pair of long spines and area IV with a median one in male	**2**
2	Distributed in Hong Kong, the anterior margin of prosoma with a row of nine tubercles, and the femur of pedipalp ventrally with nine seta-tipped tubercles.	***P. rapax***
–	Distributed in Hainan Island, the anterior margin of prosoma with a row of 12 tubercles, and the femur of pedipalp ventrally with 13 seta-tipped tubercles	***P. columnarius***

### Plistobunus jaegeri sp. nov.

Taxon classificationAnimaliaOpilionesEpedanidae

FD72E71B-8D28-526A-A55E-F4D9B3BCF655

http://zoobank.org/C5408C12-5A2B-4FEE-A38B-91571B8989E4

Figures 32–39 , 40–54 , 55–61 , 62–67

#### Type material.

***Holotype*** male (SMF-CJM7060): THAILAND: Ubon Ratchathani Province: Khong Chiam District, Pha Taem NP (No.28), 15°23.94'N 105°30.49'E, alt. 185 m, 5 November 2010, S. Dashdamirov leg. ***Paratype.*** One female (SMF-CJM7062): THAILAND: Nong Khai Province: Sangkhom District, Tham Tip Waterfall (No. 23), 18°7.57'N 102°11.16'E, alt. 198 m, 3 November 2010, S. Dashdamirov leg.

#### Diagnosis.

**Figures 32–39.**
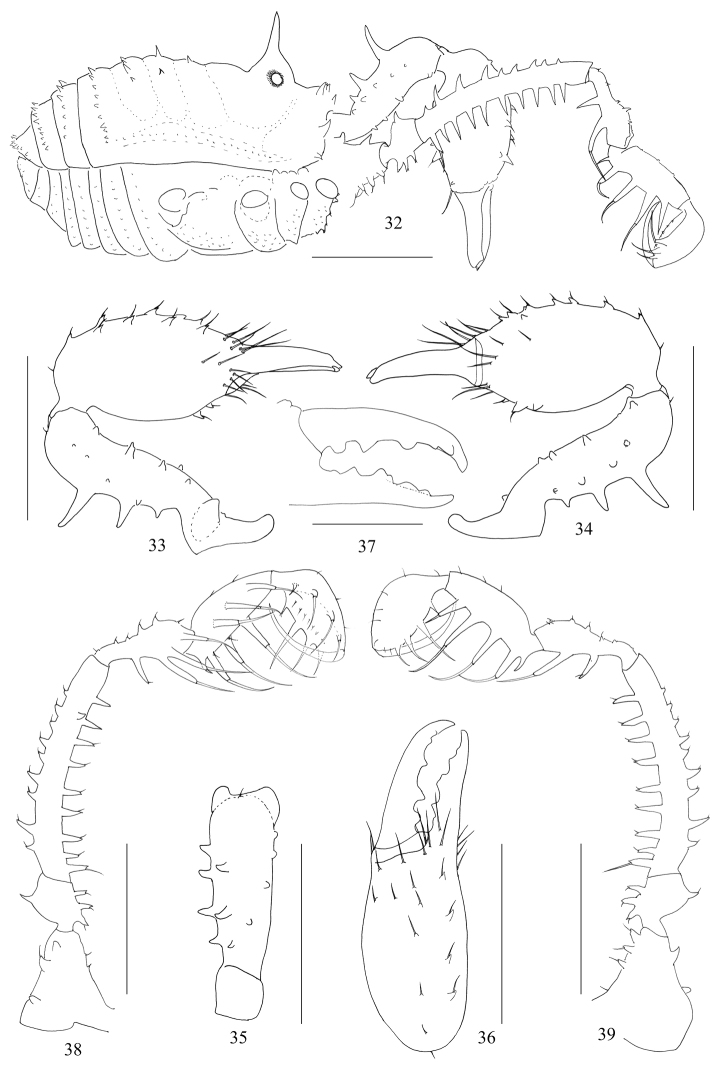
*Plistobunus jaegeri* sp. nov., male holotype **32** body, lateral view **33** left chelicera, medial view **34** same, lateral view **35** left basichelicerite, dorsal view **36** left cheliceral hand, frontal view **37** left cheliceral fingers, frontal view **38** left pedipalp, medial view **39** same, lateral view. Scale bars: 1 mm (**32–36, 38, 39**), 0.5 mm (**37**).

The dorso-distal margin (blue in Fig. 57a) of ventral plate much higher than the dorso-distal margin (purple in Fig. 57a) of pars basalis, the ventral and dorsal side of distal margin of pars basalis of penis not in the same level (cf. Fig. 57b). Dorsal opisthosomal areae II and IV without long spines in male (Figs 32, 63). The pedipalpal femur medio-distally with one seta-tipped tubercle, and the male patella with one short tubercle at base instead of unarmed in female (Figs 38, 52).

#### Etymology.

The new species is dedicated to Dr. Peter Jäger, Germany, an esteemed arachnologist. He is a co-founder of the Asian Society of Arachnology (ASA) and due to his efforts, arachnology in Asia has taken a large step forward.

#### Description.

**Figures 40–54.**
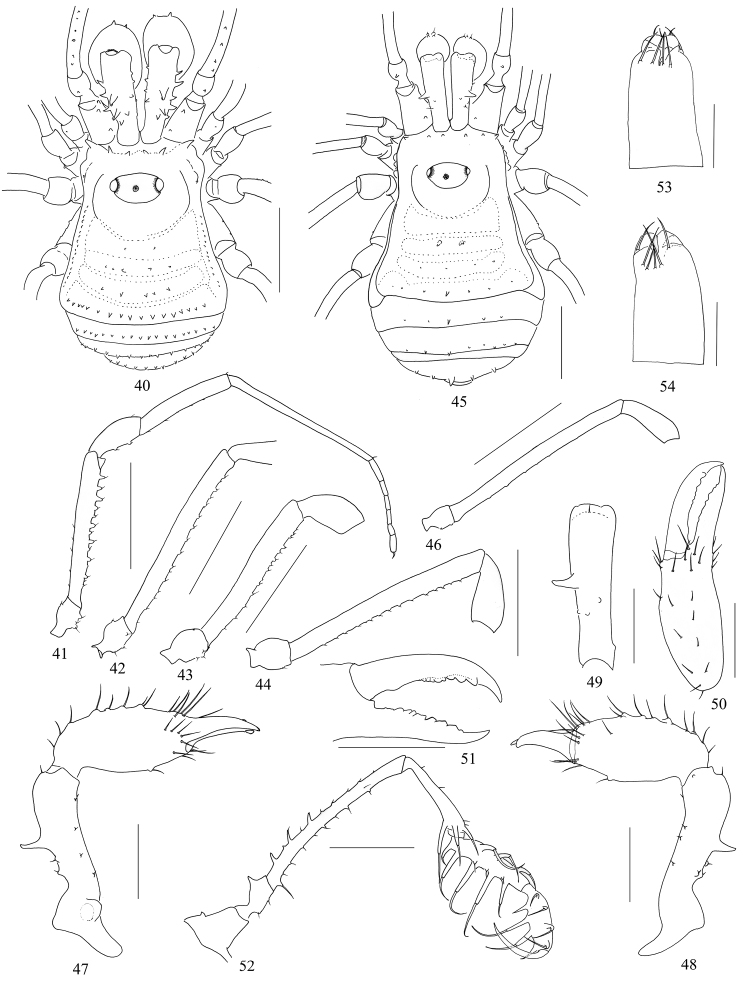
*Plistobunus jaegeri* sp. nov. male (Figs 40–44, holotype) and female (Figs 45–54, paratype) **40, 45** body, dorsal view **41** right leg I, retrolateral view **42–44, 46** femur of right legs, retrolateral view **42** leg II **43** leg III **44** leg IV **46** leg I **47** left chelicera, medial view **48** same, lateral view **49** left basichelicerite, dorsal view **50** left cheliceral hand, frontal view **51** left cheliceral fingers, frontal view **52** left pedipalp, medial view **53** ovipositor, ventral view **54** same, dorsal view. Scale bars: 1 mm (**40–46, 52**), 0.5 mm (**47–51**), 0.25 mm (**53, 54**).

**Male** (holotype) habitus as in Figs 32, 40, 62, 63. Coloration (Figs 62, 63): entire body dorsally rusty yellow with brown patches; median area of prosoma with brown reticulations in front of the ocularium; on each side behind ocularium brown outlines; both lateral ridges of the prosomal and opisthosomal scuta with dark brown stripes; opisthosomal areae I–III with light brown outlines; areae IV and V with a dark transverse band; free tergites with three large dark brown blotches; venter concolorous with the dorsum; chelicerae, pedipalps and legs rusty yellow, reticulated with light to dark brown.

***Dorsum*** (Figs 40, 62). Dorsal scutum trapezoid in shape, widest portion of body at area IV, the abdomen broadly rounded posteriorly. Anterior margin of prosoma with six tubercles. Ocularium oval, removed from anterior border of scutum by 0.40 mm, armed with a long median upright spine. The anterior margin of opisthosomal area I strongly bowed and the borders of other areae slightly bowed. Opisthosomal area I with a median pair of seta-tipped tubercles; areae II and III with similar median tubercles in addition to a few smaller ones on each side; areae IV and V and free tergites with a transverse row of seta-tipped tubercles.

***Venter*** (Fig. 64). Coxae with tubercular surface. Coxa I with enlarged seta-tipped tubercles. Coxae II–IV with minute seta-tipped tubercles. Genital operculum with a few seta-tipped granules. Free sternites with a row of small seta-tipped tubercles. Spiracles clearly visible.

***Chelicerae*** (Figs 33–37). Basichelicerite elongate, its dorsal side centrally with three spines, distal one the longest and proximal one the shortest; the lateral side with a row of four medium-sized spines; on the medial side four seta-tipped tubercles spirally arranged, in addition with a basal protuberance; ventral side with eight seta-tipped tubercles. Cheliceral hand considerably broad, pro-dorsally with some seta-tipped tubercles; three seta-tipped tubercles on the ventral side. Fingers strong, inner edges toothed (Fig. 37): moveable finger and fixed finger with five stumpy teeth.

***Pedipalps*** (Figs 38, 39). Coxa dorsally and ventrally each with three seta-tipped tubercles. Trochanter ventrally with three, dorsally with one seta-tipped tubercles. Femur ventrally with a row of nine; dorsally with a row of nine seta-tipped tubercles; medio-distally with one seta-tipped tubercle. Patella ventro-mesally and ventro-ectally each with two seta-tipped tubercles. Tibia ventro-mesally with three and ventro-ectally with four and ventrally with one seta-tipped basal tubercle. Tarsus with four seta-tipped tubercles on ventral side. Tarsal claw curved, longer than tarsus.

***Legs*** (Figs 41–44). Trochanters I–IV ventrally with a few small seta-tipped granules. Femora I–IV ventrally with longitudinal row of many seta-tipped tubercles; size on femur I enlarged and on femora II–IV reduced, on femur IV inconspicuous. Patella I ventrally with two seta-tipped granules. Tarsi III and IV with smooth double claws, without scapulae. Distitarsi I and II two-jointed. The remaining leg segments unarmed. Tarsal formula (I–IV): 6(2)/15(2)/6/7.

**Figures 55–61.**
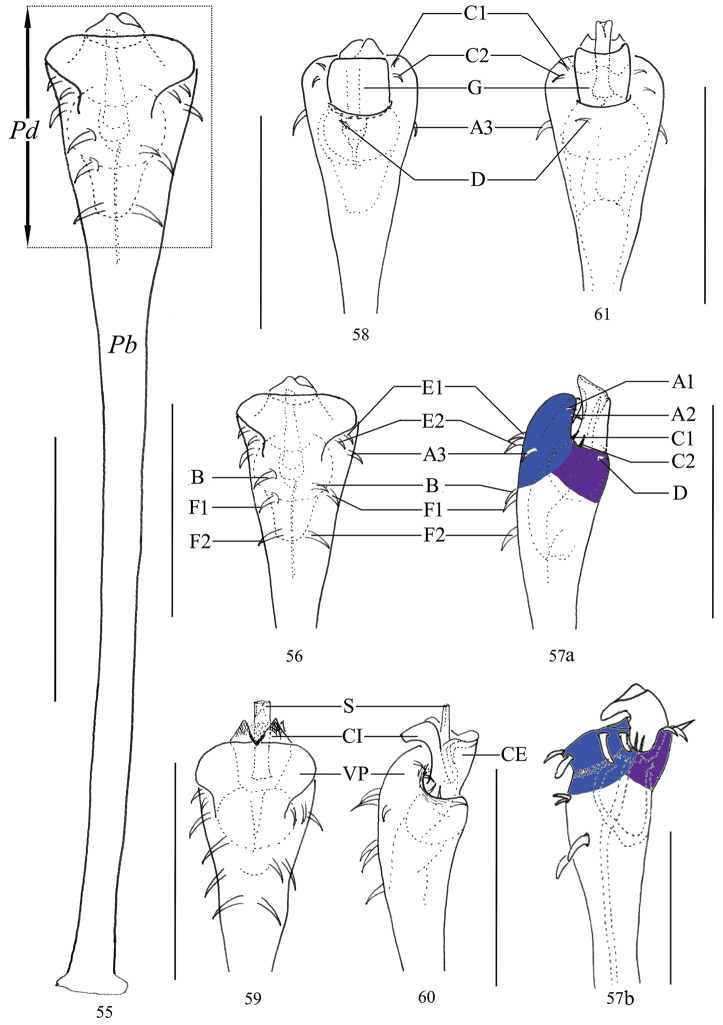
*Plistobunus jaegeri* sp. nov., genitalia of male holotype (not included Fig. 57b) **55** penis, ventral view **56** pars distalis of penis, ventral view **57a** same, lateral view **58** same, dorsal view **57b** pars distalis of penis, lateral view (*Plistobunus columnarius*Lian et al., 2011) **59** pars distalis of penis (everted), ventral view **60** same, lateral view **61** same, dorsal view. Abbreviations: **CE** capsula externa **CI** capsula interna **G** glans **Pb** pars basalis **Pd** pars distalis **S** stylus **VP** ventral plate. Scale bars: 0.25 mm.

***Penis*** (Figs 55–61). The shaft slender, parallel-sided, enlarged towards glans, distal end broadest. Distal part of truncus, i.e., glans area, almost trapezoid (ventral view, Figs 55, 56, 59), and dorsally without an indentation on the distal margin (Fig. 56, cf. Fig. 19). Glans partially sunken into dorsally depressed portion of pars distalis of penis, its tip slightly extending the distal margin (blue in Fig. 57a) of the ventral plate. The distal extension of capsula externa of semi-circular shape (Fig. 60). The distal extension of capsula interna fused into a V-shaped structure (ventral view, Fig. 59). Stylus columnar, flat distally (lateral view, Fig. 60). Spination asymmetrical, in position a single seta D; spination symmetrical, one pair of setae B, two pairs of setae C, E, and F, and three pairs of setae A.

**Female** (Figs 45–54, 65–67). In general appearance similar to the male. Scutum more widely trapezoid and wider than that of male in the posterior margin. Opisthosomal area II with a median pair of spines, area IV with a median spine (Fig. 66). Chelicera smaller and with reduced tubercles, the dorsal surface of basichelicerite centrally with one spine, the ventral surface of cheliceral hand with one seta-tipped tubercle, inner edges of finger toothed as illustrated (Fig. 51). Pedipalpal (Fig. 52) femur ventrally with a row of six, dorsally with three conspicuous seta-tipped tubercles, medio-distally without any seta-tipped tubercle; patella with three seta-tipped tubercles. The seta-tipped tubercles on femur I (Fig. 46), as well as femora II–IV inconspicuous. Tarsal formula (I–IV): 7(2)/18(2)/7/8.

***Ovipositor*** (Figs 53, 54). On ventral side with four and on dorsal side with six setae.

#### Measurements.

Male holotype (female paratype). Body 2.40 (3.53) long, 1.94 (2.08) wide at the widest portion, scutum1.68 (2.23) long. Ocularium 0.33 (0.32) long, 0.61 (0.56) wide. Proximal chelicerae 1.24 (1.11) long, 0.35 (0.30) wide; second 1.80 (1.50) long, 0.60 (0.42) wide; distal 0.76 (0.76) long, 0.19 (0.16) wide. Pedipalpus claw 0.60 (0.79) long. Penis 0.94 long. Measurements of pedipalpus and legs as in Tables 3 and 4.

**Table 3.** *Plistobunus jaegeri* sp. nov. Measurements of the pedipalp and legs of the male holotype, as length/width.

	Trochanter	Femur	Patella	Tibia	Metatarsus	Tarsus	Total
Pedipalp	0.43/0.25	1.44/0.20	0.68/0.23	0.70/0.27		0.51/0.31	3.76
Leg I	0..27/0.23	1.33/0.16	0.48/0.23	0.91/0.16	1.53/0.08	0.87/0.06	5.39
Leg II	0.30/0.26	1.80/0.15	0.56/0.22	1.43/0.15	2.03/0.06	1.71/0.05	7.83
Leg III	0.36/0.33	1.33/0.23	0.44/0.29	1.00/0.20	1.72/0.11	0.99/0.07	5.84
Leg IV	0.35/0.31	1.95/0.20	0.61/0.23	1.34/0.21	2.28/0.12	1.18/0.09	6.71

**Table 4.** *Plistobunus jaegeri* sp. nov. Measurements of the pedipalp and legs of the female paratype, as length/width.

	**Trochanter**	**Femur**	**Patella**	**Tibia**	**Metatarsus**	**Tarsus**	**Total**
Pedipalp	0.46/0.26	2.19/0.17	1.00/0.25	0.91/0.26		0.82/0.24	5.38
Leg I	0.27/0.21	1.46/0.15	0.51/0.22	1.29/0.11	2.28/0.06	1.03/0.04	5.84
Leg II	0.33/0.23	2.64/0.13	0.55/0.22	2.42/0.13	3.20/0.06	2.75/0.06	11.89
Leg III	0.34/0.36	2.08/0.18	0.56/0.31	1.44/0.19	2.91/0.10	1.14/0.06	8.47
Leg IV	0.41/0.33	3.24/0.21	0.63/0.30	1.86/0.20	4.10/0.12	1.49/0.08	11.73

#### Habitat.

The specimens were collected by hand under stones in forest.

#### Distribution.

Known only from the type locality, the Pha Taem National Park in the Ubon Ratchathanl Province, Thailand.

**Notes.** The genus *Plistobunus* was known by two species restricted to China, i.e., *P. rapax* Pocock, 1903 (type species, Hong Kong) and *P. columnarius*Lian et al., 2011 (Hainan Island). The most distinctive characters of these two species are a pair of long dorsal spines on opisthosomal area II and a median long spine on area IV. However, these characters are not conspicuous in the male of *P. jaegeri* sp. nov. (Figs 32, 63).

**Figures 62–67.**
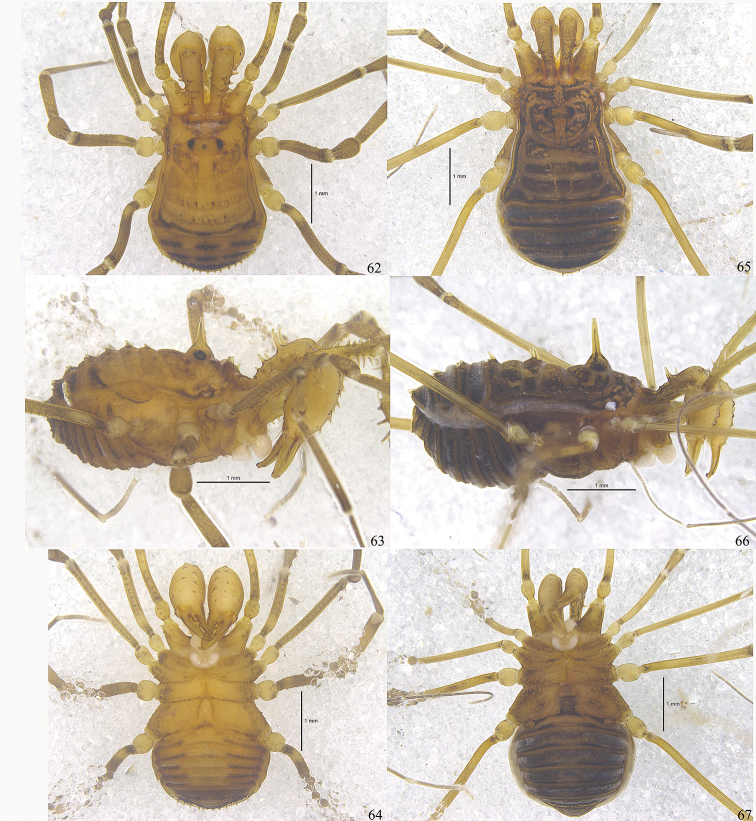
Photographs of male (Figs 62–64, holotype) and female (Figs 65–67, paratype) of *Plistobunus jaegeri* sp. nov. **62, 65** body and parts of appendages, dorsal view **63, 66** body and parts of appendages, lateral view **64, 67** body and parts of appendages, ventral view. Scale bars: 1 mm.

### Toccolus

Taxon classificationAnimaliaOpilionesEpedanidae

Roewer, 1927

96C57940-2E4B-50AD-925C-00163A7B2C4B

Toccolus Roewer 1927: 203; 1938: 102; Kury 2008: 62.

#### Type species.

*Toccolus minimus* Roewer, 1927, by monotypy.

#### Extended diagnosis.

Medium-sized epedanines (1.40–3.21 mm) with a median spine on the ocularium. Opisthosomal areae unarmed except for some seta-tipped tubercles. The basichelicerite of chelicerae armed with small or enlarged tubercles. Pedipalpal femur ventrally with five or six seta-tipped tubercles, and distally with two or three tubercles on medial side. Pedipalpal patella ventro-mesally with two tubercles, and ventro-ectally with one tubercle. Distitarsus of leg I with two segments.

#### Included species.

*Toccolus minimus* Roewer, 1927, *Toccolus chibai* Suzuki, 1976, *Toccolus globitarsis* Suzuki, 1969, *Toccolus javanensis* Kury, 2008, and *Toccolus kuryi* sp. nov.

#### Distribution.

Thailand, Java, Malaysia, Vietnam.

### Key to the currently known species of *Toccolus*

**Table:** 

1	Basichelicerite with small tubercles (the height almost as long as its width)	***T. minimus* (Vietnam)**
–	Basichelicerite with enlarged tubercles (the height much longer than its width)	**2**
2	Ocularium with a long median spine	***T. chibai* (Malaysia)**
–	Ocularium with a short median spine	**3**
3	Basichelicerite dorsally with four conspicuous spines	***T. javanensis* (Java)**
–	Basichelicerite dorsally with three conspicuous spines	**4**
4	Pedipalpal femur ventrally with five seta-tipped tubercl	***T. globitarsis* (Thailand)**
–	Pedipalpal femur ventrally with six seta-tipped tubercles	***T. kuryi* sp. nov. (Thailand)**

### Toccolus globitarsis

Taxon classificationAnimaliaOpilionesEpedanidae

Suzuki, 1969

A3917DDD-C08F-56DB-94CE-FB2AB10FE526

Figures 68–72 , 73–86 , 87–95 , 96–101

Toccolus globitarsis Suzuki 1969b: 91–96, fig. 9–11, pls 5–6.

#### Type specimens

(not examined). ***Holotype***, male: THAILAND: Paktong Cha, Nakhon Ratchasima Province: 24 November – 10 December 1963. ***Paratypes***: three females, same collection data as the holotype. All specimens were collected by H. Watanabe.

The type-specimens are all deposited in the Zoological Laboratory, Faculty of Science, Hiroshima University.

#### Additional material examined.

One male, one female and one juvenile (SMF-CJM7053), THAILAND: Chaiyaphum Province: Phakdi Chumphon Distr., near cave Wat Tham Kaeo (No. 15), 15°58.50'N 101°24.66'E, 350 m alt. 1 November 2010, S. Dashdamirov leg.

#### Redescription.

**Figures 68–72.**
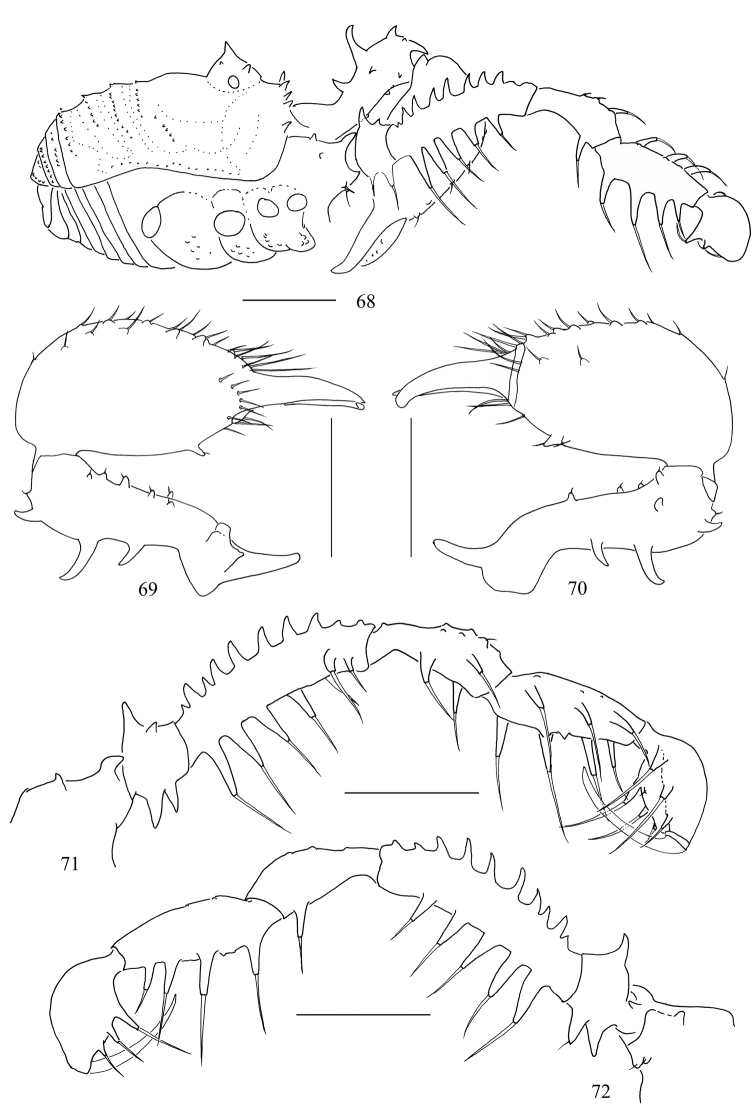
*Toccolus globitarsis* Suzuki, 1969. Male **68** body, lateral view **69** left chelicera, medial view **70** same, lateral view **71** left pedipalp, medial view **72** same, lateral view. Scale bars: 1 mm.

**Figures 73–86.**
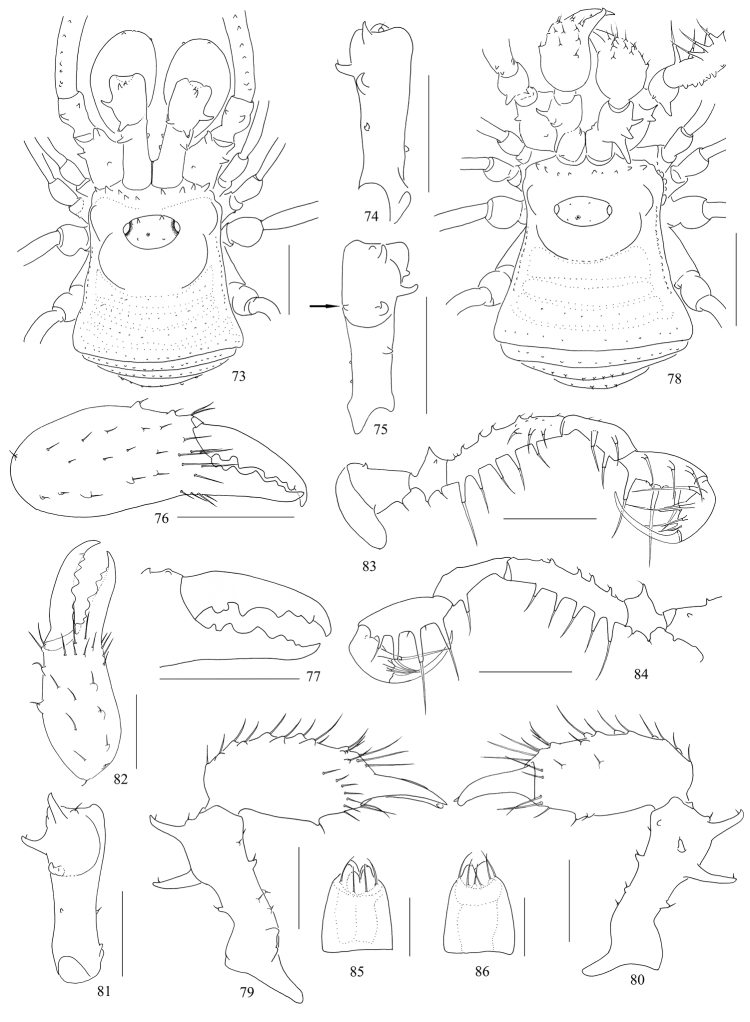
*Toccolus globitarsis* Suzuki, 1969, male (Figs 73–77) and female (Figs 78–86) **73, 78** body, dorsal view **74, 75** basichelicerite, dorsal view **74** left **75** right **76** left cheliceral hand, frontal view **77** left cheliceral fingers, frontal view **79** left chelicera, medial view **80** same, lateral view **81** left basichelicerite, dorsal view **82** left cheliceral hand, frontal view **83** left pedipalp, medial view **84** same, lateral view **85** ovipositor, ventral view **86** same, dorsal view. Scale bars: 1 mm (**73–78, 83–84**), 0.5 mm (**79–82**), 0.25 mm (**85–86**).

**Male** habitus as in Figs 68, 73, 96, 97. Coloration (Figs 96, 97): body light yellow with brown patches; median area of prosoma with brown reticulations; both lateral ridges of scutum with brown stripes; opisthosomal areae I–III each with a transverse band of brown markings; area IV and all free tergites with a cross series of three brown flecks; all coxae yellow with brown reticulations distally; free sternites each with brown bands, bands somewhat paler on the central portion; chelicerae and pedipalps with reticulated irregular markings; trochanters pale yellow, femur, patella, tibia, and metatarsus with black reticulations, tarsus lighter.

***Dorsum*** (Figs 73, 96). Scutum nearly parallel-sided, slightly trapezoid, the widest portion at scutal area IV, and abdomen bluntly pointed posteriorly. Prosoma with a row of nine sharp pointed tubercles along anterior margin (Fig. 73); otherwise whole dorsum smooth. Ocularium oval, removed from anterior border of scutum by 0.33 mm, armed with a conspicuously shorter median spine than the height of ocularium (lateral view), and two small tubercles above the eyes. The posterior area of ocularium on prosoma raised and broadly rounded. The anterior margin of opisthosomal area I bowed and the borders of other areae slightly bowed. Opisthosomal areae I–IV with a row of seta-tipped tubercles and a longitudinal row of similar tubercles on the lateral margins. Free tergites with a transverse row of seta-tipped tubercles each, anal operculum with scattered granules.

***Venter*** (Fig. 98). Coxae with tubercular surface, coxa I with enlarged seta-tipped tubercles. Coxae II–IV with minute tubercles. Genital operculum and free sternites with seta-tipped granules. Spiracles clearly visible.

***Chelicerae*** (Figs 69–70, 74–77). Basichelicerite elongate and armed with three long, curved spines dorsally (Figs 69, 70); one similar spine distally on the ectal side (Figs 70, 74); six seta-tipped tubercles scattered over the ventral side (Fig. 69); medial side with a basal protuberance (Fig. 69). Tubercle on left basichelicerite more medially than on right one (arrowed in Fig. 75). Cheliceral hand considerably widened, pro-dorsally with some seta-tipped tubercles, the inner ones much larger than the outer ones (Fig. 76); ectally with two seta-tipped tubercles at the subdistal portion (Figs 70, 76); one enlarged seta-tipped tubercle and a smaller one on the ventral side (Fig. 70). Fingers strong, inner edges toothed (Fig. 77): moveable finger with five teeth, the proximal one square, the other four pointed; fixed finger with six crested teeth.

***Pedipalps*** (Figs 71, 72). Coxa dorsally with three seta-tipped tubercles, one strong distal, one subdistal more exteriorly placed, one blunt proximal; ventrally with two seta-tipped tubercles. Trochanter ventrally with two setiferous tubercles, dorsally with an enlarged one and a small one. Femur ventrally with a row of five setiferous tubercles of which the longest one close to the base; dorsally with a row of ten setiferous tubercles of which the distal two inconspicuous; distally on medial side with two setiferous tubercles. Patella ventro-mesally with two setiferous and ventro-ectally with one setiferous tubercles. Tibia ventro-mesally with three and ventro-ectally with four setiferous tubercles. Tarsus on ventral side para-median with three setiferous tubercles each. Tarsal claw curved, longer than tarsus.

***Legs*.** All segments unarmed, nearly smooth. Legs I–II slender, legs III–IV much stronger. Femora I–III not curved, almost straight, femur IV slightly curved. Distitarsi I and II with two tarsomeres. Distitarsi III and IV without scopula, only two bare claws present. Tarsal formula (I–IV): 7(2)/16(2)/6/7.

**Figures 87–95.**
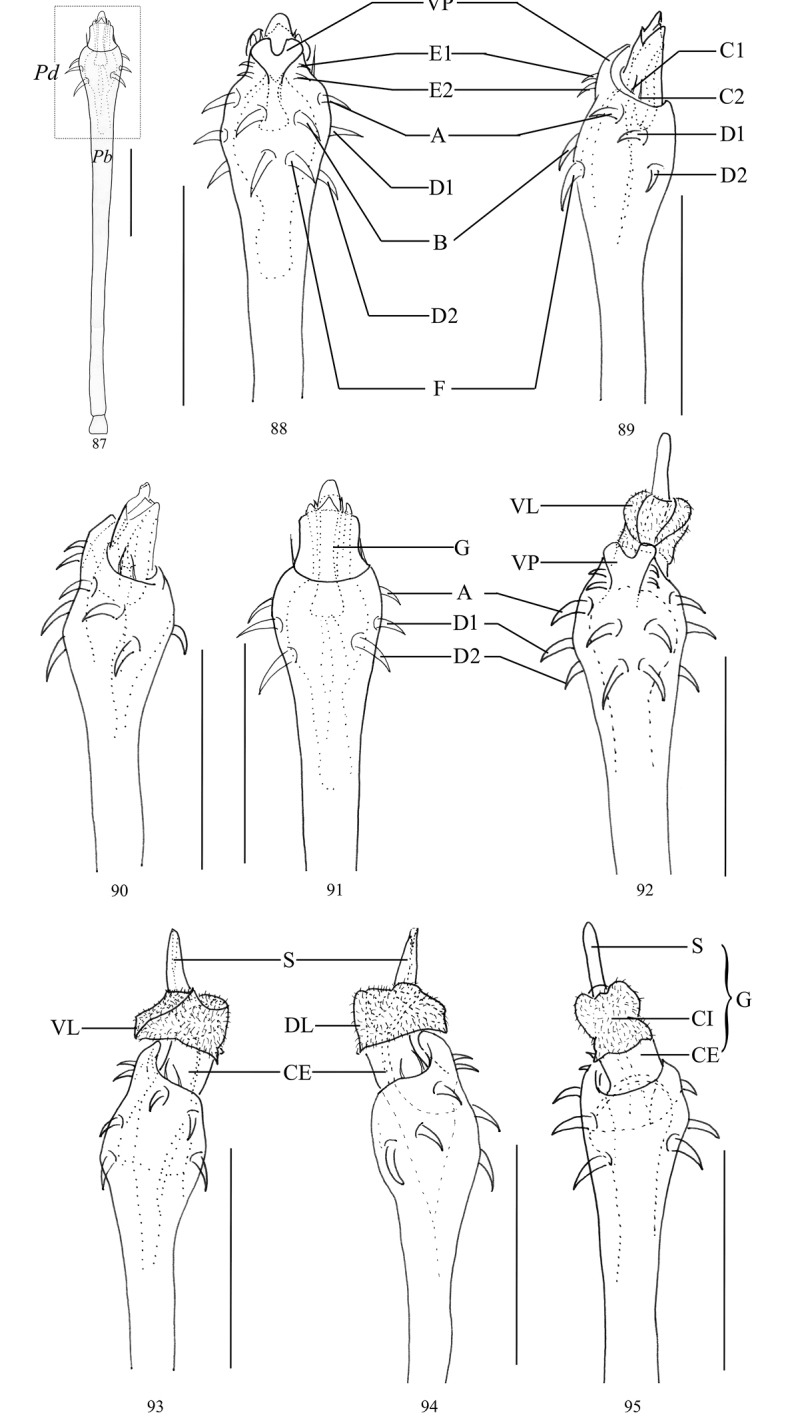
*Toccolus globitarsis* Suzuki, 1969, genitalia of male **87** penis, dorsal view **88** pars distalis of penis, ventral view **89, 90** same, lateral view **91** same, dorsal view **92** pars distalis of penis(everted), ventral view **93, 94** same, lateral view **95** same, dorsal view. Abbreviations: **CE** capsula externa **CI** capsula interna **DL** dorsal lobe **G** glans **Pb** pars basalis **Pd** pars distalis **S** stylus **VP** ventral plate **VL** ventral lobe. Scale bars: 0.25 mm.

***Penis*** (Figs 87–95). Shaft slender, nearly parallel-sided and only slightly distended, then more pronounced towards apical portion (pars distalis). Ventral surface of pars distalis formed by ventral plate conspicuously widened distally, and at its distal end with a deep and rounded incision (Fig. 88). Glans partially sunken into dorsally depressed portion of pars distalis and extending the distal margin of the ventral plate (Figs 88–90). Capsula externa cylindrical. Capsula interna inside with a dense coat of fur-like microtrichia, including ventral lobe and dorsal lobe; everted ventral lobe ventrally extended beyond the ventral plate, and folded on both sides; everted dorsal lobe embracing the capsula externa, and with two small triangular projections on the sides of the distal margin of pars distalis. Stylus curled by the membrane forming irregular shape. Spination symmetrical. One pair of setae A, B, and F. Two pairs of setae C1–2, D1–2, and E1–2 (Figs 88–92).

#### Female

(Figs 78–86, 99–101). Generally similar to male except abdomen slightly wider than in male (Figs 78, 99). The median spine on ocularium greatly enlarged and ocularium with two reduced tubercles above each eye. Chelicerae not enlarged but of normal shape, with a slight difference in inner edges of cheliceral finger (Fig. 82). Femora of pedipalpi dorsally with a row of five setiferous tubercles, distally on medial side with three setiferous tubercles. Tarsal formula (I–IV): 6(2)/15(2)/6/7.

***Ovipositor*** (Figs 85, 86). Ventral surface with four setae and dorsal surface with six setae.

#### Measurements.

Male (female): body 2.89 (2.41) long, 2.35 (2.10) wide at widest portion, scutum 2.27 (1.80) long. Ocularium 0.45 (0.32) long, 0.77 (0.62) wide. Proximal chelicerae 1.60 (1.01) long, 0.50 (0.36) wide; second 2.56 (1.52) long, 0.92 (0.51) wide; distal 1.15 (0.74) long, 0.25 (0.14) wide. Pedipalpal claw 0.95 (0.87) long. Penis 1.15 long. Measurements of pedipalp and legs as in Tables 5 and 6.

**Table 5.** *Toccolus globitarsis* Suzuki, 1969. Measurements of the pedipalp and legs of the male, as length/width.

	**Trochanter**	**Femur**	**Patella**	**Tibia**	**Metatarsus**	**Tarsus**	**Total**
Pedipalp	0.60/0.41	1.68/0.34	0.81/0.39	1.07/0.40		0.83/0.50	4.99
Leg I	0.30/0.24	1.68/0.18	0.57/0.23	1.13/0.15	1.97/0.06	1.09/0.04	6.74
Leg II	0.34/0.27	2.31/0.15	0.67/0.23	1.89/0.13	2.88/0.08	1.97/0.05	10.06
Leg III	0.39/0.37	1.97/0.25	0.62/0.37	1.30/0.23	2.23/0.11	0.83/0.06	7.34
Leg IV	0.41/0.39	2.40/0.27	0.69/0.36	1.64/0.24	2.86/0.15	1.17/0.08	9.17

**Table 6.** *Toccolus globitarsis* Suzuki, 1969. Measurements of the pedipalp and legs of the female, as length/width.

	**Trochanter**	**Femur**	**Patella**	**Tibia**	**Metatarsus**	**Tarsus**	**Total**
Pedipalp	0.48/0.34	1.23/0.28	0.77/0.30	0.80/0.34		0.64/0.34	3.92
Leg I	0.28/0.21	1.25/014	0.52/0.20	1.04/0.12	1.66/0.06	0.91/0.05	5.66
Leg II	0.33/0.24	1.71/0.13	0.56/0.21	1.07/0.11	2.45/0.06	1.82/0.05	8.57
Leg III	0.31/0.34	1.47/0.19	0.55/0.31	1.15/0.20	1.90/0.11	0.65/0.06	6.03
Leg IV	0.33/0.34	1.45/0.20	0.63/0.30	1.51/0.19	2.62/0.08	1.19/0.06	7.73

#### Habitat.

The specimens were collected in litter and under stones.

#### Distribution.

Thailand (Nakhon Ratchasima Province, Chieng Mai Province, Chaiyaphum Province).

**Figures 96–101.**
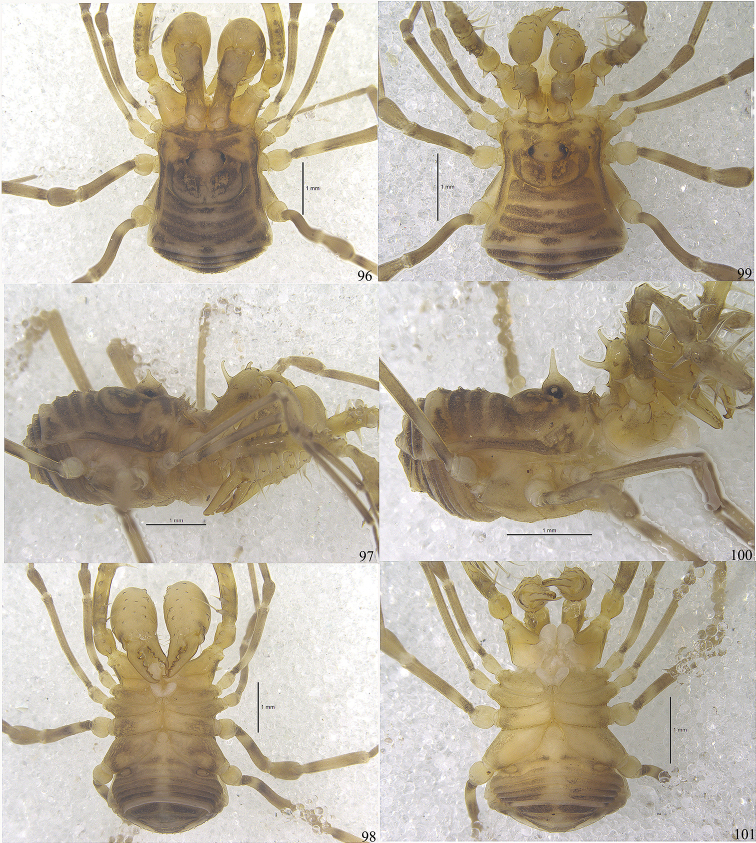
Photographs of male (Figs 96–98) and female (Figs 99–101) of *Toccolus globitarsis* Suzuki, 1969. **96, 99** body and parts of appendages, dorsal view **97, 100** body and parts of appendages, lateral view **98, 101** body and parts of appendages, ventral view. Scale bars: 1 mm.

### Toccolus kuryi sp. nov.

Taxon classificationAnimaliaOpilionesEpedanidae

936A11B7-63CA-5827-8751-BFC806B77B27

http://zoobank.org/F49E92F7-8667-435F-A842-438280CEE8CA

Figs 102–109 , 110–117 , 118–123 , 124–129

#### Type material.

***Holotype*** male (SMF-CJM7065): THAILAND: Loel Province: Nang Hin District, near cave Wat Tham Malohan (No.18a), 17°06.47'N 101°52.69'E, alt. 362 m, 2 November 2010, S. Dashdamirov leg. ***Paratype*.** One female (SMF-CJM7063): THAILAND: Nong Khai Province: Sangkhom District, Tham Tip Waterfall (No. 23), 18°07.57'N 102°11.16'E, alt. 198 m, 3 November 2010, S. Dashdamirov leg.

#### Diagnosis.

The distal part of glans nearly triangular (ventral and dorsal views, Figs 119, 121) and the tip curved ventrally (lateral view, Fig. 120). Basichelicerite dorsally with three spines (Figs 103, 104, 112, 113). Pedipalpal femur ventrally with a row of six setiferous tubercles, the longest one at the base (Figs 108, 116).

#### Etymology.

The specific name is a patronym in honor of Dr. Adriano B. Kury, a well-known arachnologist from Brazil. A. Kury (2000) created a website of Opiliones and built a platform for acquiring knowledge accessible for everyone. He redefined the family Epedanidae to include four subfamilies (Kury 1993).

#### Description.

**Figures 102–109.**
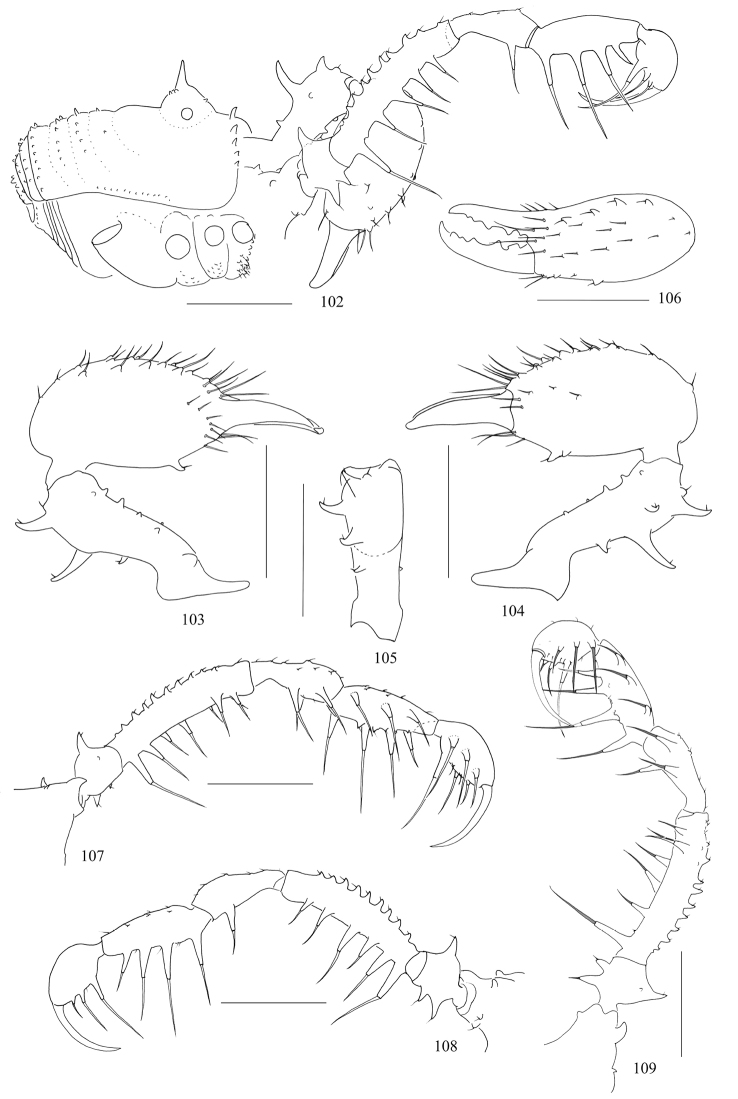
*Toccolus kuryi* sp. nov., male holotype **102** body, lateral view **103** left chelicera, medial view **104** same, lateral view **105** left basichelicerite, dorsal view **106** left cheliceral hand, frontal view **107** left pedipalp, medial view **108** same, lateral view **109** right pedipalp, medial view. Scale bars: 1 mm.

**Figures 110–117.**
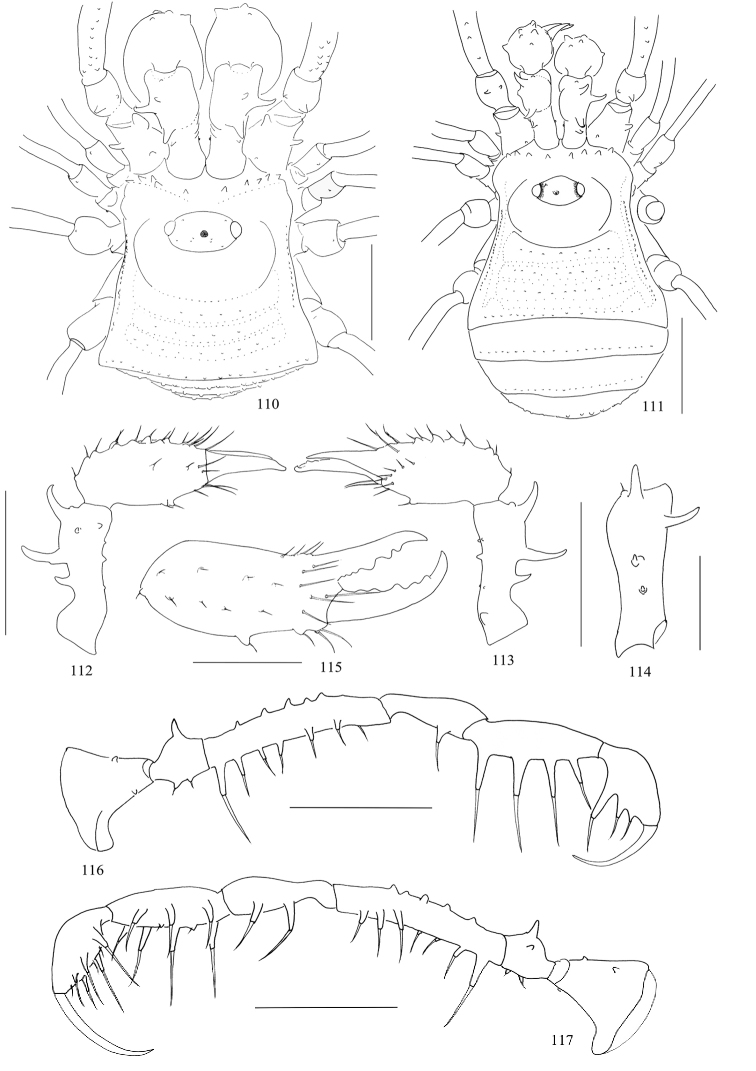
*Toccolus kuryi* sp. nov., male (Fig. 110, holotype) and female (Figs 111–117, paratype) **110, 111** body, dorsal view **112** right chelicera, lateral view **113** same, medial view **114** right basichelicerite, dorsal view **115** right cheliceral hand, frontal view **116** right pedipalp, lateral view **117** same, medial view. Scale bars: 1 mm (**110–113, 116, 117**), 0.5 mm (**114, 115**).

**Male** (holotype) habitus as in Figs 102, 110, 124–126. Coloration (Figs 124–126): body light yellow with brown patches; median area of prosoma with brown reticulations; both lateral ridges of scutum with brown stripes; opisthosomal areae I–III each with a transverse brownish band; area IV with a series of three brown flecks; all free tergites brown; all coxae yellow with brown reticulations distally; free sternites each with brown bands, these somewhat paler on the central portion; chelicerae and pedipalps reticulated; trochanters pale yellow, femur, patella, tibia and metatarsus with black reticulations, tarsus lighter.

***Dorsum*** (Figs 110, 124). Scutum trapezoid in appearance, both parts nearly parallel-sided, the widest portion of body at opisthosomal area IV, posterior end of opisthosoma bluntly rounded. Prosoma with a row of ten sharply pointed tubercles along anterior margin. Dorsal scutum smooth. Ocularium oval, removed from anterior border of prosoma scutum by 0.38 mm, armed with a conspicuous short median spine of about the height of ocularium (lateral view), four small tubercles above both eyes. The area behind ocularium raised, and the posterior margin of prosoma smoothly rounded. The anterior margin of opisthosomal area I markedly bowed out, borders of other scutal areae slightly bowed out. Opisthosomal areae I–IV with a row of seta-tipped tubercles each and a longitudinal row of similar tubercles on left and right lateral margins. Free tergites with a transverse row of seta-tipped tubercles and anal operculum with scattered granules.

***Venter*** (Fig. 126). Coxae with tubercular surface, coxa I additionally with enlarged seta-tipped tubercles. Coxae II–IV with minute tubercles. Genital operculum and free sternites with seta-tipped granules. Spiracles clearly visible.

***Chelicerae*** (Figs 103–106). Basichelicerite elongate, armed with two long, curved spines, one short spine dorsally; another one distally on the ectal side; six seta-tipped tubercles scattered over the ventral side; medial side with a basal protuberance (Fig. 103). Cheliceral hand considerably widened, pro-dorsally with few seta-tipped tubercles, the inner ones larger than the outer ones; ectally with two seta-tipped tubercles at the subdistal portion; one enlarged seta-tipped tubercle and a smaller one on the ventral side. Fingers strong, inner edges toothed (Fig. 106): moveable finger with five teeth, the proximal one cut rectangularly, the other four pointed; fixed finger with five pointed teeth.

***Pedipalps*** (Figs 107–109). Coxa dorsally with three seta-tipped tubercles, one strong distal, one sub-distal exterior to it, and a blunt one situated proximally; ventrally with two seta-tipped tubercles. Trochanter ventrally with two setiferous tubercles and dorsally with an enlarge one and a small one. Femur ventrally with a row of five (right pedipalp, Fig. 102) or six (left pedipalp, Fig. 108) setiferous tubercles, the longest one at the base of the row; dorsally with a row of ten setiferous tubercles, distal one and proximal one inconspicuous; disto-medially side with two setiferous tubercles. Patella ventro-mesally with two and ventro-ectally with one setiferous tubercles. Tibia ventro-mesally with three and ventro-ectally with four setiferous tubercles. Tarsus with three setiferous tubercles each ventro-ectally and ventro-medially. Tarsal claw massive, curved, longer than tarsus.

***Legs*.** All segments unarmed, nearly smooth. Legs I–II slender, legs III–IV strong. Femora I–III not curved, almost straight, femur IV slightly curved. Distitarsi I and II with two tarsomeres. Distitarsi III and IV without scopula and with two unpectinated claws. Tarsal formula (I–IV): 7(2)/16(2)/7/7.

**Figures 118–123.**
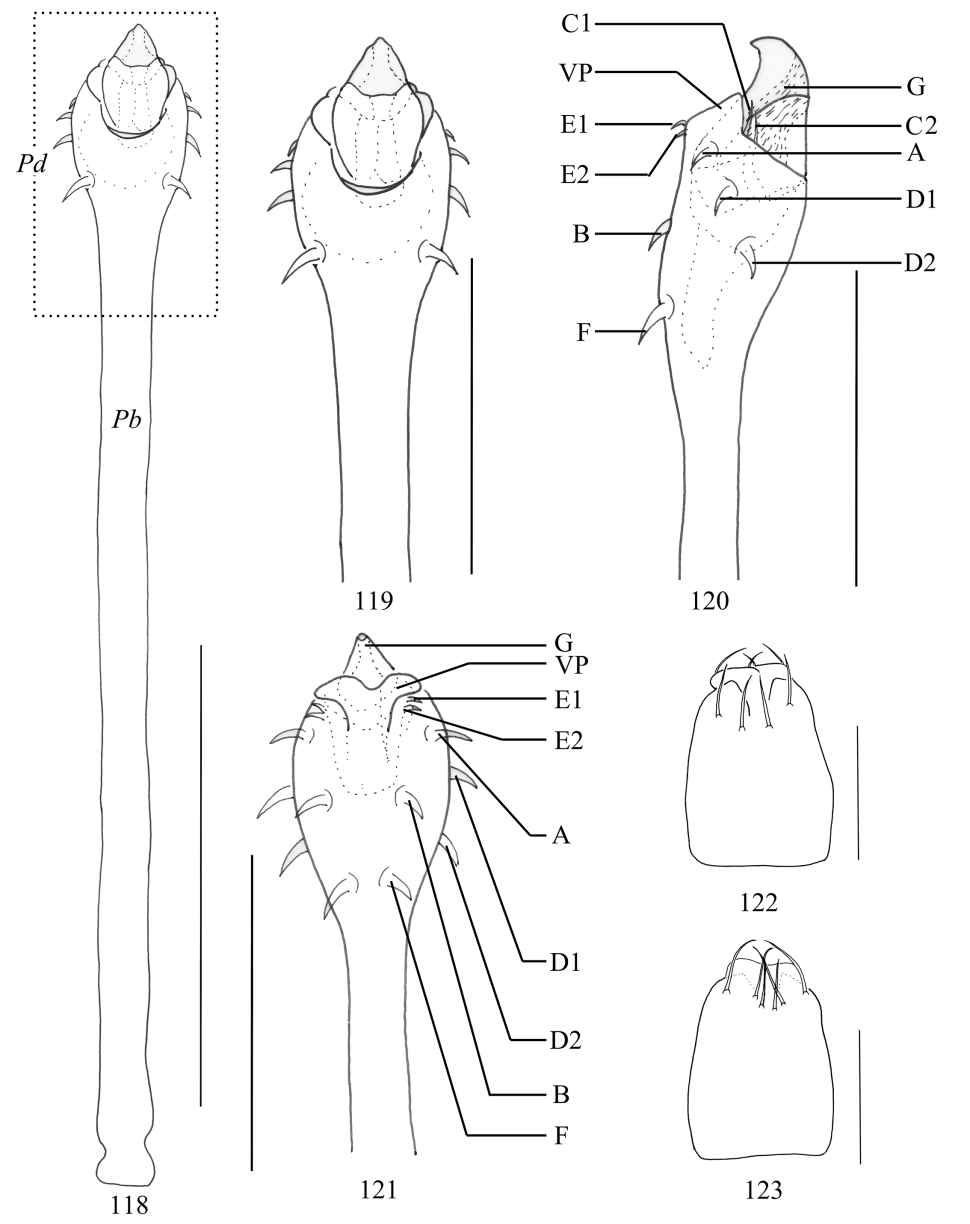
*Toccolus kuryi* sp. nov., genitalia of male holotype **118** penis, dorsal view **119** pars distalis of penis, dorsal view **120** same, lateral view **121** same, ventral view **122** ovipositor, ventral view **123** same, dorsal view. Abbreviations: **G** glans **Pb** pars basalis **Pd** pars distalis **VP** ventral plate. Scale bars: 0.5 mm (**118**), 0.25 mm (**119–123**).

***Penis*** (Figs 118–121). Shaft slender, nearly parallel-sided, apical part (pars distalis) distended. Ventral plate conspicuously constricted apically, and dorsally with deep indented near the glans. Glans partially sunken into dorsal depressed portion of pars distalis and extended the distal margin of the ventral plate (Fig. 120). The distal part of glans almost triangular (ventral and dorsal views, Figs 119, 121), the tip curved ventrally. Spination symmetrical. One pair of setae A, B, and F. Two pairs of setae C1–2, D1–2, and E1–2 (Figs 120, 121).

#### Female

(SMF-CJM7063) (Figs 111–117, 122, 123, 127–129). Body nearly oval. Granulation and spination of body similar to the male (Figs 111, 127). Prosoma with a row of eight sharp pointed tubercles along anterior margin (Fig. 111). Surface of dorsum smooth. Ocularium with only one conspicuous tubercle near the left eye except for the long median spine. Chelicerae of normal shape, not enlarged, slight difference in inner edges of cheliceral finger (Fig. 115). Femora of pedipalpi ventrally with six and dorsally with a row of five setiferous tubercles, disto-medially with three setiferous tubercles. Tarsal formula (I–IV): 6(2)/15(2)/7/7.

***Ovipositor*** (Figs 122, 123). Ventral side with four, dorsal side with six setae.

**Measurements.** Male (female): body 2.27 (2.76) long, 2.08 (1.96) wide at widest portion, scutum 1.95 (1.79) long. Ocularium 0.41 (0.30) long, 0.69 (0.51) wide. Proximal chelicerae 1.24 (0.84) long, 0.47 (0.31) wide; second 2.20 (1.37) long, 0.78 (0.45) wide; distal 0.97 (0.62) long, 0.22 (0.14) wide. Pedipalp claw 0.82 (0.79) long. Penis 1.02 long. Measurements of pedipalp and legs as in Tables 7 and 8.

**Table 7.** *Toccolus kuryi* sp. nov. Measurements of the pedipalp and legs of the male holotype, as length/width.

	**Trochanter**	**Femur**	**Patella**	**Tibia**	**Metatarsus**	**Tarsus**	**Total**
Pedipalp	0.56/0.43	1.56/0.30	0.98/0.32	0.95/0.35		0.70/0.38	4.75
Leg I	0.35/0.24	1.60/0.17	0.52/0.23	1.12/0.13	1.94/0.06	1.06/0.04	6.59
Leg II	0.34/0.25	2.15/0.14	0.59/0.21	1.86/0.12	2.74/0.05	1.94/0.04	9.62
Leg III	0.42/0.29	1.86/0.22	0.61/.032	1.30/0.21	2.21/0.12	1.03/0.07	7.43
Leg IV	0.48/0.35	2.32/0.24	0.63/0.32	1.76/0.21	2.71/0.11	1.15/0.05	9.05

**Table 8.** *Toccolus kuryi* sp. nov. Measurements of the pedipalp and legs of the female paratype, as length/width.

	**Trochanter**	**Femur**	**Patella**	**Tibia**	**Metatarsus**	**Tarsus**	**Total**
Pedipalp	0.42/0.34	1.28/0.23	0.78/0.26	0.87/0.27		0.66/0.29	4.01
Leg I	0.24/0.22	1.24/0.15	0.48/0.19	0.93/0.11	1.54/0.06	0.64/0.04	5.71
Leg II	0.22/0.19	1.72/0.13	0.52/0.19	1.51/0.10	2.27/0.05	2.46/0.04	8.70
Leg III	0.26/0.30	1.35/0.18	0.53/0.27	1.07/0.19	1.79/0.09	0.76/0.08	5.76
Leg IV	0.32/0.28	1.67/0.19	0.56/0.25	1.39/0.17	2.48/0.08	1.13/0.07	7.55

#### Habitat.

The specimens were collected under stones (SMF-CJM7065) and under *Acacia* bark and rotten stumps (SMF-CJM7063).

#### Distribution.

Thailand (Loel Province, Nong Khai Province).

**Figures 124–129.**
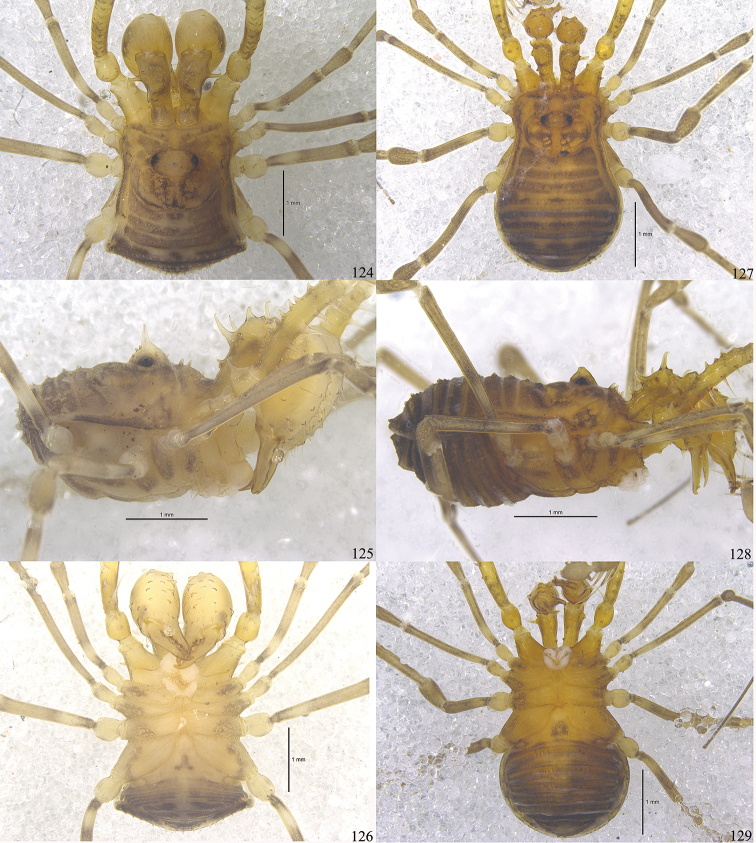
Photographs of male (Figs 124–126, holotype) and female (Figs 127–129, paratype) of *Toccolus kuryi* sp. nov. **124, 127** body and parts of appendages, dorsal view **125, 128** body and parts of appendages, lateral view **126, 129** body and parts of appendages, ventral view. Scale bars: 1 mm.

## Discussion

According to the present results, the typical members of Epedanidae (Acrobuninae Roewer, 1912; Sarasinicinae Roewer, 1923; Epedaninae Sørensen, 1886; and Dibuninae Roewer, 1912) possess similar external morphology, e.g., “the greatly elongate pedipalps, high erect spine on eye tubercle, fused scutal areas I–II etc” (Lian et al. 2008: 58). However, the morphology of the male genitalia of epedanids is in fact highly diverse.

**Figures 130–141.**
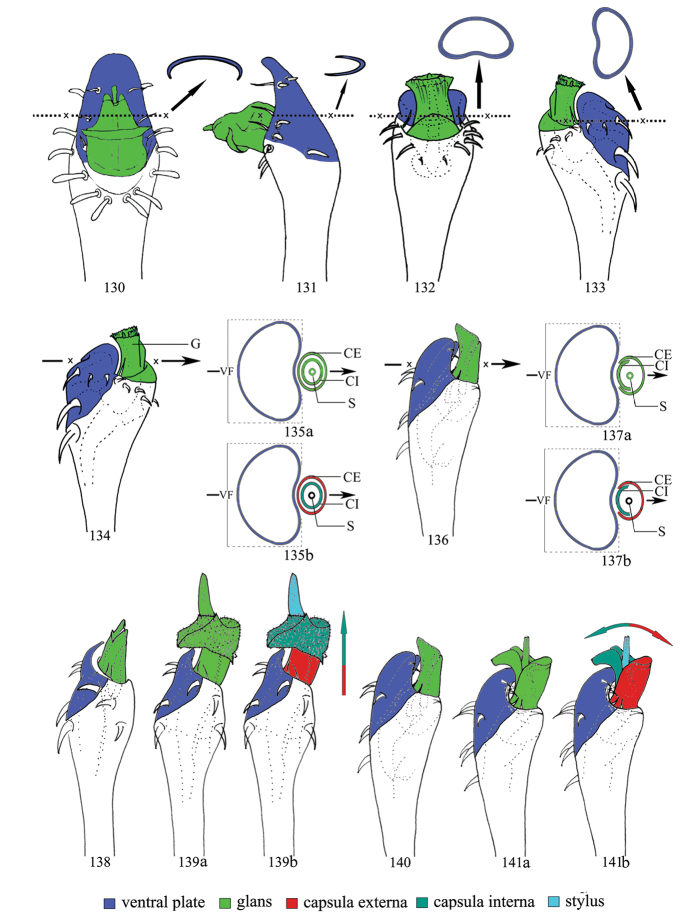
Schematic drawings contrasting the morphology of pars distalis (Pd) of male genitalia in different representatives of the Epedanidae**130, 131***Epedanellus tuberculatus* Roewer, 1911 (arrow indicates the cross-section of ventral plate at the marked point, adapted from Suzuki, 1973) **130** ventral view **131** lateral view **132, 133***Lobonychium palpiplus* Roewer, 1938 (arrow indicates the cross-section of ventral plateat the marked point, adapted from Zhang & Martens, 2018) **132** dorsal view **133, 134** lateral view **135a, b** cross-section through ventral plate and glans at the marked point **136, 137, 140, 141***Plistobunus jaegeri* sp. nov. **136** lateral view **137** cross-section through ventral plate and glans at the marked point **138, 139***Toccolus globitarsis* Suzuki, 1969 **138** lateral view **139a, b** expanded, lateral view (arrow indicates the movement of capsula interna in 139b) **140** lateral view **141a, b** expanded, lateral view (arrow indicates the movement of capsula interna and capsula externa in 141b).

The penis can be classified at least into two different types by the shape of the ventral plate. One type of ventral plate is well defined by a single plate (Figs 130, 131) which is shovel-shaped (Fig. 130) and usually is C-shaped in cross-section. Its distal border greatly extends the penis by more than the length of the glans (Fig. 130).

The other type of ventral plate is called a ventral frame (Zhang and Martens 2018) and is not sharply defined by a three-dimensional structure (Figs 132–137). This plate is circularly shaped in cross-section (Fig. 135) and is almost completely covered by the glans (Fig. 132). The distal margin of ventral plate is straight or concave.

The structures of the capsula externa and the capsula interna fall into at least two groups. One group is characterized by a saccular and circular capsula externa and capsula interna (Figs 134, 135a, b). The other group is defined by an arc-shaped capsula externa and capsula interna (Figs 136, 137a, b). Until now it is not clear if these differences may have an impact on epedanid taxonomy and systematics.

We assume that during copulation glans and stylus are exposed by internal hemolymph pressure. The movement causes the glans sclerites to expand under the internal pressure in two different ways. The trajectory of saccular and circular capsula externa and capsula interna is unidirectional and they are extruded in straight prolongation of the entire penis cane (Figs 138, 139a, b). In contrast, the movement of penes with the arc-shaped capsula externa and capsula interna appears opposite and they move up and down according of the internal pressure applied (Figs 60, 140, 141a, b; e.g., Lian et al. 2011: 48, fig. 31).

## Supplementary Material

XML Treatment for Euepedanus

XML Treatment for Euepedanus dashdamirovi

XML Treatment for Plistobunus

XML Treatment for Plistobunus jaegeri

XML Treatment for Toccolus

XML Treatment for Toccolus globitarsis

XML Treatment for Toccolus kuryi
